# Intravenous Tranexamic Acid Versus Topical Aminocaproic Acid: Which Method Has the Least Blood Loss and Transfusion Rates?

**DOI:** 10.5435/JAAOSGlobal-D-18-00072

**Published:** 2018-11-07

**Authors:** Zachary C. Lum, Martin A. C. Manoukian, Christopher S. Pacheco, Alexander J. Nedopil, Mauro Giordani, John P. Meehan

**Affiliations:** From the Department of Orthopaedics, Adult Reconstruction Section, University of California: Davis Medical Center, Sacramento, CA.

## Abstract

**Introduction:**

Since the advent of antifibrinolytics, blood transfusions and their associated complications in total joint arthroplasty have decreased. Few studies have compared different antifibrinolytic types with respect to blood loss and transfusion rates. We sought to compare the blood loss and transfusion rates between epsilon-aminocaproic acid (EACA), tranexamic acid (TXA), and control.

**Methods:**

A total of 564 patients underwent primary total hip or total knee arthroplasty at our institution. Patients were divided into 3 groups: 183 EACA, 204 TXA, and 177 control. Patient demographics, hemoglobin, transfusion rates, and blood loss were collected.

**Results:**

Patient preoperative variables were similar. The control group had a mean estimated blood loss (EBL) of 1.48 L, with 51 units of packed red blood cells (pRBCs) given and 14.7% of patients receiving a blood transfusion. The EACA group had an EBL of 1.33 L, with 20 pRBCs given and 10.9% of patients receiving a transfusion. The TXA group had an EBL of 1.05 L, with 3 pRBCs transfused in 0.98% of patients. Compared with the control group, blood loss (*P* = 0.0014; *P* < 0.0001), number of pRBCs given (*P* = 0.007; *P* < 0.0001), and number of patients transfused (*P* = 0.012; *P* < 0.0001) were significantly lower in the EACA and TXA groups, respectively. TXA had significantly lower blood loss (*P* < 0.0001), lower number of tranfusions (*P* = 0.005), and less patients transfused (*P* = 0.003) compared with EACA.

**Conclusion:**

Our study reports lower blood loss, transfusion rates, and number of patients needing transfusion with both EACA and TXA in the setting of total joint arthroplasty. When comparing between EACA and TXA, TXA had lower blood loss, transfusion rates, and number of patients requiring transfusion.

Blood loss and transfusions in total joint arthroplasty have been revolutionized by the arrival of antifibrinolytics. Before their use, studies had routinely reported transfusion rates of 10% to 24%, with direct and indirect costs up to $1,200.^1,2,3,4,5^ With addition of these medications, topical or intravenous, current transfusion rates have decreased and can range from 1% to 10%.^1,2,3,4,5^

Tranexamic acid (TXA) and epsilon-aminocaproic acid (EACA) belong to the lysine analog class of antifibrinolytic agents. They have similar mechanisms of action, with TXA demonstrating a 6- to 10-fold increased affinity in binding plasminogen compared with EACA.^6,7^ Because of its high affinity, TXA has mostly replaced EACA as the predominant lysine analog used for major orthopaedic procedures.

Few studies have compared different antifibrinolytic types with respect to blood loss and transfusion rates. We sought to compare whether EACA or TXA had the least amount of blood loss and transfusion rates with a control group for comparison. We hypothesized that although there may be a difference in blood loss, there would be no difference with regard to transfusion rates or units of blood given.

## Methods

From January 2008 to June 2016, a retrospective chart review of 564 patients who underwent primary total hip (THA) or total knee arthroplasty (TKA) was performed by 2 surgeons at our institution. Patients were sequentially selected via the CPT code for primary THA (ie, 27130) or primary TKA (ie, 27447). For our control cohort, patients were selected from an older cohort before hospital approval of antifibrinolytic administration. Patients were excluded for preoperative anemia, for revision surgery, osteoarthritis from a secondary disease (eg, inflammatory, posttraumatic), previous surgery to the surgical side, active infection, or history of bleeding or clotting disorders.

Patients were divided into 3 groups. The EACA group received 5 g/100 mL of saline mixture applied topically. This mixture was applied before tourniquet release and left in the wound for at least 1 minute. The TXA group received 1 g/10 mL IV or 3 g/100 mL topically. Patients received primarily IV TXA, unless the patient had atrial fibrillation, cardiac stents, previous history of venous thromboembolic (VTE) disease, or stroke. Ten patients received topical TXA.

Perioperative management was the same for the EACA and TXA groups. For these 2 cohorts, VTE prophylaxis postoperatively included aspirin 325 mg daily or warfarin with goal INR 1.5 to 2.0. Patients primarily received aspirin unless they have significant VTE risk factors such as previous recent VTE within 6 months or pulmonary embolism with decompensation. The control group received surgery earlier in the date range and was administered enoxaparin or warfarin with the same INR goals. This was before our anticoagulation committee's approval of aspirin as a form of VTE prophylaxis. The medial parapatellar approach was used for all TKAs, and the posterolateral approach was used for all THAs. One surgeon performed the gap balancing technique for TKAs and used primarily EACA for hemostasis; the other surgeon performed measured resection and used primarily TXA. Both surgeons are fellowship trained in adult reconstruction at the same fellowship.

Patient demographics, including age, sex, weight, height, and body mass index (BMI), were collected. Preoperative hemoglobin and subsequent postoperative hemoglobins were obtained until discharge. Transfusions rates and blood loss were calculated using the Gross equation (estimated blood loss [EBL] = estimated blood volume [EBV] × [Hct_0_ − Hct_f_]/Hct_AV_).

## Statistical Analysis

Patients receiving no antifibrinolytic drug were considered the control group for analysis, whereas the patients receiving an antifibrinolytic were categorized in the TXA or EACA group. Continuous variables were described using mean values and SDs and compared using the Student *t*-test. Categoric variables were shown as frequency and percentages and were compared using the chi-square test. Preoperative analysis for age, female sex, preoperative hemoglobin, and BMI was performed. Postoperative hemoglobins were trended and EBV was predicted using sex and height, and entered into the EBL equation using the Gross calculation for blood loss (EBL = EBV × [Hb_0_ − Hb_f_]/Hb_AV_).

## Results

A total of 564 patients underwent THA or TKA, with 177 patients receiving no antifibrinolytic, 183 in the EACA cohort, and 204 in the TXA cohort. Patient preoperative variables were similar (Table 1). The average age of the control group was 61.9 years (21 to 90 years), EACA group was 62.7 years (25 to 88 years), and the TXA group was 65 years (24 to 85 years). No difference was found between the control and EACA groups (*P* = 0.4625); however, the TXA group was significantly older than both the EACA and control groups (*P* = 0.02; *P* = 0.0026).

**Table 1:**
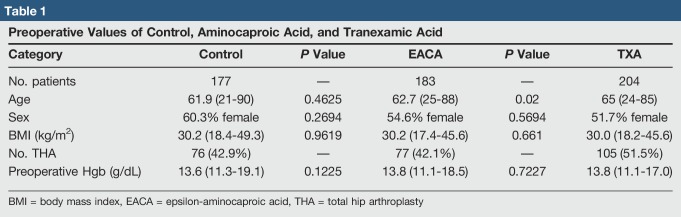
Preoperative Values of Control, Aminocaproic Acid, and Tranexamic Acid

The percentage of female in the control, EACA, and TXA groups were 60.3%, 62.7%, and 51.7%, respectively, and demonstrated no difference between the control and EACA groups (*P* = 0.2694), control and TXA groups (*P* = 0.0899), and EACA and TXA groups (*P* = 0.5694). BMI in the control, EACA, and TXA cohorts were 30.2 kg/m^2^ (18.4 to 49.3 kg/m^2^), 30.2 kg/m^2^ (17.4 to 45.6 kg/m^2^), and 30.0 kg/m^2^ (18.2 to 45.6 kg/m^2^), respectively, and demonstrated no difference between the control and EACA groups (*P* = 0.9619), control and TXA groups (*P* = 0.7137), and EACA and TXA groups (*P* = 0.5694). Preoperative hemoglobin in the control group measured 13.6 g/dL (11.3 to 19.1), EACA group 13.8 g/dL (11.1 to 18.5), and TXA group 13.8 g/dL (11.1 to 17.0). No difference was found between the control and EACA groups (*P* = 0.1225), control and TXA groups (*P* = 0.0526), and EACA and TXA groups (*P* = 0.7227).

The control group had an EBL of 1,480 mL, with 51 units of packed red blood cells (pRBCs) given and 14.7% of patients receiving a blood transfusion (Table 2). The EACA group had an EBL of 1,330 mL with 20 pRBCs given and 10.9% of patients receiving a transfusion. The TXA group had an estimated 1,050 mL blood loss, with 3 units of pRBCs transfused in 0.98% of patients. Compared with the control group, blood loss (*P* = 0.0014; *P* < 0.0001), number of pRBCs given (*P* = 0.007; *P* < 0.0001), and number of patients transfused (*P* = 0.012; *P* < 0.0001) were significantly lower in the EACA and TXA groups, respectively. Thus, both antifibrinolytics resulted in lower blood loss and transfusion rates.

**Table 2:**
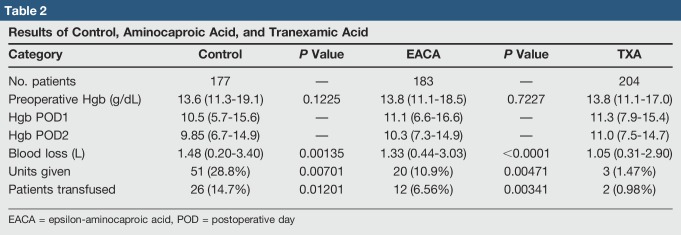
Results of Control, Aminocaproic Acid, and Tranexamic Acid

When comparing the EACA versus TXA groups, the TXA group had significantly less blood loss (1,330 mL versus 1,050 mL; *P* < 0.0001), lower number of tranfusions (10.9% versus 1.47%; *P* = 0.005), and less patients transfused (6.56% versus 0.98%; *P* = 0.003) compared with the EACA group.

## Discussion

Antifibrinolytics have significantly lowered the transfusion rates and associated transfusion-related complications in total joint arthroplasty. Many studies have reported lower transfusion rates with intravenous or topical TXA compared with placebo.^1,2,3,4,6-8^ In addition, studies involving EACA have also reported lower transfusion rates in TJA.^1,2,3,4,8^ Our results agree that both antifibrinolytics EACA and TXA significantly decrease transfusion rates compared with placebo control (*P* = 0.012; *P* < 0.0001), respectively.

Our cohort of 564 patients divided into 3 groups of 184 EACA, 204 TXA, and 177 control reported less blood loss and blood transfusions in the antifibrinolytics versus control. In addition, we were surprised to have found a significantly lower blood loss and transfusion rate in favor of TXA versus EACA.

Few studies directly compare blood loss and transfusion rates between EACA and TXA. Churchill et al^3^ retrospectively compared transfusion rates between EACA, TXA, and a control group in 2,922 TKAs. They reported that significantly fewer patients received blood transfusion in the EACA and TXA groups (2.8%, *P* < 0.0001; 3.2%, *P* < 0.0001) compared with the control group (10.8%). On their comparison, they did not report any difference in transfusion rates between the two antifibrinolytics (*P* = 0.822). Their group also retrospectively investigated transfusion rates between the 2 antifibrinolytics and a control in 1,799 THAs.^2^ They also found significantly lower transfusion rates in the EACA group (6.8%; *P* < 0.0001) and TXA group (9.7%; *P* < 0.0001) compared with the control group (24.7%), with no difference in transfusion rates between the two antifibrinolytics (*P* = 0.074). Although our results confirmed that both antifibrinolytics significantly lowered transfusion rates, we reported a significant difference in transfusion rates between EACA and TXA. One of the discrepancies noted was their preoperative variables. In their TKA cohort, the TXA cohort was significantly older (65.8 versus 63.9; *P* = 0.001), had higher number comorbidities (*P* = 0.0096), and had significantly lower preoperative hemoglobin than EACA (*P* < 0.0001), all surrogates for increased blood loss and transfusion rates. In their THA cohort, the TXA cohort had significantly lower preoperative hemoglobin than EACA (*P* = 0.02), a risk factor for higher transfusion rates and blood loss. These confounding variables may skew the results of their data in favor of EACA. Although our number of comorbidities was not recorded, our preoperative hemoglobin and BMI were not different. Our age groups favored against TXA, with a higher age in TXA compared with EACA and control (65 versus 62.7 and 61.9; *P* = 0.02).

There are 2 level 1 studies comparing EACA, TXA, and placebo in TKA. Camarasa et al^8^ randomized 128 patients undergoing primary TKA to receive TXA, EACA, or placebo. Powered only to detect all antifibrinolytics combined versus control, they reported significantly lower transfusion rates and units (7.5% versus 38.3%; *P* < 0.001). They had 35 patients who received TXA and 32 patients who received EACA. When comparing between these 2 groups, 2.8% of TXA patients received a transfusion compared with 12.5% of EACA. Although this difference seems large, this was not found to be significant, possibly because of the lack of power and sample size. This scenario was mentioned in their study. Compared with our data, the overall percentages of transfused patients are similar in TXA (2.8% versus 0.98%) and EACA (12.5% versus 6.56%). Although their level 1 study could not find a difference in transfusion rates between TXA and EACA, they were not powered to detect a difference.

Boese et al^1^ performed a recent level 1 randomized control trial comparing EACA versus TXA in TKA in 194 patients. Although they reported that both arms of their study had zero transfusions, blood loss in the TXA was significantly lower than EACA at 144.2 mL (*P* = 0.031), although this was deemed to be clinically not significant. One important fact is that their power analysis to detect differences was much lower than their recruitment study size, which may result in lower numbers reported.

The strengths of our study are our relatively larger sample size at a single tertiary level 1 center. Our surgeons used the same approaches with similar perioperative protocols. We found no difference in preoperative variables. In addition, our average hospital length of stay was longer than all the other comparison studies, which means we may have captured more accurate data regarding true blood loss.

Our study had several limitations. Besides being a retrospective study chart review, one surgeon used primarily one antifibrinolytic, EACA, which may place our results at risk of bias. Although most perioperative protocols were standardized, the anticoagulation was not. Some patients received aspirin; others received warfarin, which can affect wound drainage and potentially perioperative blood loss. This scenario was difficult to control for, and all of the previously cited EACA versus TXA comparison studies did not account for this variable as well. In addition, most patients received intravenous TXA compared with topical EACA. Although other studies have demonstrated no difference between topical and intravenous antifibrinolytics, the route of administration was not the same in all cases. Last, we grouped THA and TKA together because both arthroplasty types can have differing transfusion rates. Although our TXA cohort had a higher percentage of THAs, this would have strengthened our study because THA has an increased risk of transfusion (51.5% versus 42.1%). Last, we used 5 g of EACA, whereas some studies used 100 mg/kg or 7 g; we used 1 g of TXA, whereas some studies used 10 mg/kg or 2 doses, possibly resulting in underdosing. Interpretation of these results may suggest that a higher EACA dosing is necessary to get a similar clinical benefit compared with TXA.

## Conclusion

Antifibrinolytics in total joint arthroplasty have revolutionarily decreased blood loss, transfusion rates, and their associated complications. The decision to use one is obvious, however which type is not clear. We reported TXA to have less blood loss, transfusion rates, and number of transfusions given compared with EACA. These data dispute other studies that demonstrate no difference in blood loss between the two medications. Additional studies with higher sample sizes may be required to discern whether a difference exists between the two antifibrinolytics.
